# High-Multiplex Aptamer-Based Serum Proteomics to Identify Candidate Serum Biomarkers of Oral Squamous Cell Carcinoma

**DOI:** 10.3390/cancers15072071

**Published:** 2023-03-30

**Authors:** Sebastian Blatt, Peer W. Kämmerer, Maximilian Krüger, Rambabu Surabattula, Daniel G. E. Thiem, Simon T. Dillon, Bilal Al-Nawas, Towia A. Libermann, Detlef Schuppan

**Affiliations:** 1 Department of Oral and Maxillofacial Surgery, University Medical Center, 55131 Mainz, Germany; 2 Institute of Translational Immunology and Research Center for Immune Therapy (FZI), University Medical Center of the Johannes Gutenberg-University, 55131 Mainz, Germany; 3 Beth Israel Deaconess Medical Center Genomics, Proteomics, Bioinformatics and Systems Biology Center, Division of Interdisciplinary Medicine and Biotechnology, Beth Israel Deaconess Medical Center, Harvard Medical School, Boston, MA 02215, USA; 4 Division of Gastroenterology, Beth Israel Deaconess Medical Center, Harvard Medical School, Boston, MA 02215, USA

**Keywords:** oral cancer, SOMAscan, proteomics, liquid biopsy, biomarker, serum, prognosis, therapy

## Abstract

**Simple Summary:**

Oral cancer is a life-threatening disease and among the ten most common cancer types. Specific protein biomarkers in the blood of these patients may allow early tumor detection, earlier intervention and help to individualize therapy resulting in improved patient survival. This exploratory study analyzed the serum proteome with a highly reliable technology to define novel, non-invasive biomarkers of oral cancer. Our results showed important differences of the serum levels of 63 proteins in oral cancer patients vs. controls and of 121 proteins discriminating between patients before and after curative surgery. Our study proves the feasibility of this approach to establish novel serum biomarkers that help to improve the treatment of patients with oral cancer.

**Abstract:**

Improved serological biomarkers are needed for the early detection, risk stratification and treatment surveillance of patients with oral squamous cell carcinoma (OSCC). We performed an exploratory study using advanced, highly specific, DNA-aptamer-based serum proteomics (SOMAscan, 1305-plex) to identify distinct proteomic changes in patients with OSCC pre- vs. post-resection and compared to healthy controls. A total of 63 significantly differentially expressed serum proteins (each *p* < 0.05) were found that could discriminate between OSCC and healthy controls with 100% accuracy. Furthermore, 121 proteins were detected that were significantly altered between pre- and post-resection sera, and 12 OSCC-associated proteins reversed to levels equivalent to healthy controls after resection. Of these, 6 were increased and 6 were decreased relative to healthy controls, highlighting the potential relevance of these proteins as OSCC tumor markers. Pathway analyses revealed potential pathophysiological mechanisms associated with OSCC. Hence, quantitative proteome analysis using SOMAscan technology is promising and may aid in the development of defined serum marker assays to predict tumor occurrence, progression and recurrence in OSCC, and to guide personalized therapies.

## 1. Introduction

Oral squamous cell carcinoma (OSCC) is a life-threatening disease and among the ten most common cancer types [1]. Therapy regimens are mainly based on surgery, which in advanced tumor stages is combined with adjuvant radiation and often with chemotherapy [2]. Depending on primary tumor staging, the overall five-year survival rate remains constantly low at approximately 40–60% [3,4]. For staging, anatomical–histological criteria such as primary tumor size (T), lymph node metastasis (N) and/or distant metastasis (M) are summarized in the TNM classification of the World Health Organization, unique for every solid tumor entity [5]. However, the classification fails to predict the progression or aggressiveness of the disease, or the response to adjuvant therapy [6,7]. In order to facilitate early tumor detection and help individualize therapeutic approaches and to improve follow-ups, additional novel and especially non-invasive biomarkers are urgently needed [8,9,10]. Liquid biomarkers that are obtained from body fluids such as serum or plasma, urine or saliva are non-invasive, easily accessible, low cost and usually highly reproducible. They are therefore of great interest as (future) diagnostic or therapy-guiding companion biomarkers [11,12]. In particular, the proteome that can represent the functionality of genetic information should be the most instructive and of key interest. Proteome analysis may also help to identify new pathological mechanisms of different cancer entities and provide important diagnostic tools for monitoring the progression and biological activity as well as therapy resistance of cancer [13]. The current state of the research of serum or plasma proteomics in OSCC has used conventional approaches, such as algorithmic analysis of differential protein expression levels of known proteins to distinguish between patients with oral cancer and controls in a cross-sectional or follow-up design, including attempts to detect early tumor recurrence, but these studies showed inadequate sensitivity and specificity [13,14,15,16].

Only recently, highly exact, unbiased, affinity-based methods for high scale proteome analysis with high sensitivity and specificity for a broad range of proteins across the entire dynamic range in complex fluids such as serum of plasma were introduced that overcame the shortcomings of conventional, even advanced, mass spectrometry technologies to detect novel biomarkers of disease [17,18]. The SOMAscan technology was introduced in 2012 for quantitative proteome analysis. Using modified DNA-aptamers that bind to defined proteins or peptides allows the quantitative detection of 1305, more recently ~7000, proteins at a 30 fM detection level in a multiplex assay format. This way, even a difficult-to-analyze proteome such as from serum or plasma can be investigated with a small sample size and high reproducibility [18,19,20,21]. DNA-aptamers exhibit a versatile set of chemical and physical modifications that endow them with protein-like and other functional groups to allow selective binding to a given target protein according to a key and lock mechanism, while non-specific binding is reduced. They are thus useful in (liquid) biomarker discovery to develop diagnostics for conditions of unmet clinical need that ultimately support the development of targeted therapies [20].

So far, serum or plasma proteomic studies with this technology have been mainly performed in patients with, for example, Duchenne muscular dystrophy, chronic renal insufficiency, Alzheimer’s disease, lung cancer, liver fibrosis, delirium, cardiovascular disease or non-alcoholic steatohepatitis, but exploratory aptamer-based serum/plasma proteomics has not been performed on patients with OSCC [20,22,23,24,25]. Therefore, the aim of this exploratory study was to assess this method to identify quantitative and qualitative patterns in the serum proteome of a small but well-defined cohort of patients with OSCC compared to healthy controls that may permit the development of ELISA tests for potential liquid biomarkers to predict OSCC occurrence, progression and recurrence.

## 2. Materials and Methods

In an exploratory pilot study, DNA-aptamer-based SOMAscan^®^ analysis was assessed in pre- and post-resection sera of well-defined patients with OSCC in comparison to an age-matched healthy control group. The study was conducted in accordance with the Declaration of Helsinki on Ethical Principles or Medical Research Involving Human Subjects and was approved by the local ethics committee of the state of Rhineland-Palatinate, Germany (approval number 2018-13201, date of approval: 9 May 2018).

### 2.1. Sample Collection

After informed consent of every included patient, serum samples were obtained during the prescheduled preoperative and follow-up routine blood tests (advanced OSCC: *n* = 6; with no tumor recurrence postoperatively within 3–6 months; each patient 2 samples; total *n* = 12; healthy control group: *n* = 4) and stored at −80 °C. In the OSCC group, the serum samples were obtained 24 h before and within 3–6 months after tumor resection, combined with adjuvant therapy such as irradiation or chemo-radiotherapy, where appropriate according to the current guidelines. In the control group, the samples were obtained shortly before dental surgery (dental implant surgery, tooth extraction). Apart from (mild) coronary heart disease, arterial hypertension or chronic venous insufficiency, no severe illnesses were reported (Table 1).

### 2.2. SOMAScan Analysis

All sample analyses were conducted with compliance to the Good Laboratory Practice (GLP). After transport, a SOMAscan^®^ analysis (SomaLogic, Boulder, CO, USA) quantifying 1305 distinct serum proteins (1305-plex) was performed using 55 μL of the anonymized serum samples at the Genomics, Proteomics, Bioinformatics and Systems Biology Center, Beth Israel Deaconess Medical Center, Boston, MA, USA, according to the manufacturer’s standard protocol and as previously described [21,26]. Briefly, modified DNA aptamers (SOMAmers) composed of a unique 40-mer sequence of single stranded DNA were employed. These DNA aptamers specifically bind proteins via a selective protein binding domain. Serum samples were distributed randomly in 96-well microtiter plates with the assay operators blinded to the anonymized samples. Cy3 fluorescently-labeled SOMAmer mixtures (immobilized through biotin to streptavidin coated beads) were added and unbound proteins subsequently washed away (See Graphical Abstract). Bound proteins were exposed to a biotinylation step, and SOMAmers (without the biotin) were thereafter liberated from the surface by exposure to ultraviolet light. Protein–SOMAmer complexes were then immobilized for a second time, through the biotinylated proteins to a streptavidin-coated surface. Following denaturation, SOMAmers were released from associated proteins and hybridized to custom micro-array chips, containing complementary oligonucleotides to each individual SOMAmer. Finally, Cy3 fluorescence intensity was measured which is proportional to the quantity of specific protein in the original sample [27,28].

### 2.3. Quantification of Biomarker Candidates

All assay results were reported in Relative Fluorescence Units (RFU). For data processing, microarray images were captured and processed with a microarray scanner and associated software. Five pooled human plasma controls and one non-protein buffer control were run in parallel with the plasma test samples. Sample-to-sample variability was further controlled by several hybridization spike-in controls. Data quality control, calibration and normalization were performed according to the manufacturer’s protocol as previously described [29]. Each sample in the study was normalized by aligning the median of each sample to a common reference. Inter-plate and inter-run calibration was performed by applying a multiplicative scaling coefficient to each SOMAmer. These scaling factors were calculated using the eight reference calibrators on each plate [28].

### 2.4. Bioinfornatics and Statistical Analysis

The mean and median fold-changes (FC) of protein expression were calculated for proteins with statistically significant different expression between OSCC cases and controls or among the OSCC cases between pre-and post-resection. Statistical significance was determined by using a *t*-test to compare SOMAscan^®^ relative fluorescence units for OSCC vs. healthy controls and a paired-*t*-test for OSCC pre- vs. post-resection. A protein was considered to be significantly dysregulated if the *p*-value for the expression between cases and controls or pre- vs. post-resection was <0.05. Heat maps or hierarchical clustering of the most significantly dysregulated proteins were generated with Morpheus (Broad Institute, Cambridge, MA, USA). Principal component analysis was performed for the top dysregulated proteins to evaluate their ability to discriminate cases from controls and ore- from post-resection OSCC using XLSTAT (Addinsoft, Long Island City, NY, USA). Box Whisker plots of median protein expression values and ROC curves were created using XLSTAT.

To assess for potential biological pathways underlying the OSCC-associated serum protein signatures and to more precisely understand the complex interactions between the differentially expressed proteins, we performed functional category, canonical pathway, interactive network, upstream regulator and regulator effect analyses of all dysregulated proteins with a *p* value < 0.05 using the Ingenuity Pathway Analysis software tool (QI-AGEN, Redwood City, CA, USA), a repository of biological interactions and functions created from millions of individually modeled relationships ranging from the molecular (proteins, genes) to organism (diseases) level.

The 63 dysregulated OSCC-associated proteins and the 121 OSCC resection-associated proteins with a *p* < 0.05 were included in a further network analysis using the STRING database version 11.5 for protein–protein functional and physical interactions, the results of which were displayed as a functional network [30]. Interactions were considered with a STRING confidence score of 0.4 or higher garnered from the “experimental” and “databases” categories. Proteins without associations to other proteins in the network were removed. A k-means clustering algorithm was performed to select the connected proteins (k-means = 3 for OSCC vs. control and k-means = 9 for OSCC ENT2 vs. ENT1). A functional description of the clusters was assigned based on a manually curated evaluation of the enriched KEGG pathway, Gene Ontology (GO), Reactome, STRING local network clusters terms and PubMed literature search.

For this exploratory study, we chose a convenience sample of six cases pre- and post-resection and four healthy controls. Given this was an exploratory study, we did not perform a sample size calculation.

## 3. Results

The characterization of the included patients is summarized in Table 1. In the control group, the patients received dental surgery for implant surgery and/or the extraction of a destroyed but not infected tooth. Of the OSCC patients, five patients were male; the mean age was 66.5 ± 4.9 years. None of the patients had any other cancers or a severe general illness but moderate comorbidities (coronary heart disease, chronic venous disease, arterial hypertension and depression) were described. Three patients needed adjuvant irradiation because of advanced tumor sizes (pT4a); one patient (pT2) was irradiated because of the aggressive phenotype of OSCC of the buccal mucosa with lymph node metastasis. Two patients had cervical lymph node metastasis, and none had distant metastases. Due to the exploratory character of the study, only a short follow up of 6 months (±2.2 months) was included. Here, no apparent tumor recurrence or metastasis occurred and no patients died.

A SOMAscan analysis of six patients with OSCC and four healthy controls identified a total of 63 significantly differentially expressed serum proteins (*p* < 0.05) that could discriminate between preoperative OSCC patients and healthy controls. The 63 up-regulated and down-regulated proteins are listed in Table 2 (refer to Supplementary Table S1 for the target and entrez gene symbol, *p* values and mean and median fold changes).

**Figure 1:**
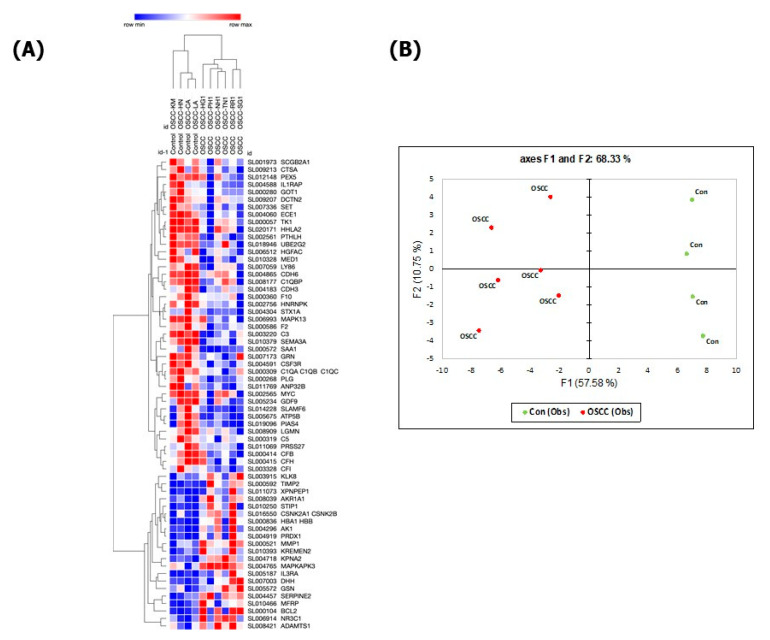
Hierarchical clustering (**A**) and Principal Component Analysis (**B**) of 63 OSCC-associated proteins that accurately discriminate between preoperative OSCC (**left** column) and healthy controls (**right** column, *p* < 0.05). Red = up-regulated, blue: down-regulated.

Hierarchical clustering of the 63 significantly differentially expressed proteins (*p* < 0.05) discriminated with 100% accuracy between the patients with OSCC and the healthy controls (Figure 1A). Several potentially functionally involved proteins were up-regulated in OSCC, such as the tissue inhibitor of metalloproteinases-2 (TIMP2), matrix metalloproteinase-1 (MMP1), peroxiredoxin 1 (PRDX1), MAP-kinase activated protein kinase 3 (MAPKAPK3), stress-inducible phosphoprotein 1 (STIP1) and glia-derived nexin (SERPINE2), while others where down-regulated, such as complement factor-I and -H, angiostatin (PLG), semaphorin-3 (SEMA3A), cadherin-3 (CDH3) and cadherin-6 (CDH6). An unsupervised principal component analysis (PCA) of all the samples using the 63 most dysregulated proteins (*p* < 0.05) (Figure 1B) also resulted in a clear separation of the OSCC cases from the healthy controls. The first principal component accounts for 57.6% of the variance, and the second principal component for 10.8% of the variance. This analysis demonstrates that the SOMAscan-derived proteomics data contain a significant component that accurately differentiates between OSCC cases compared to healthy controls.

**Figure 2:**
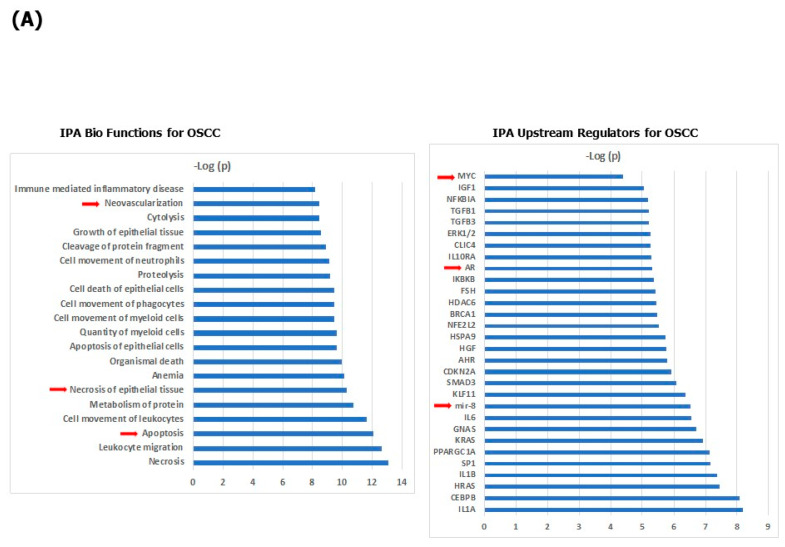

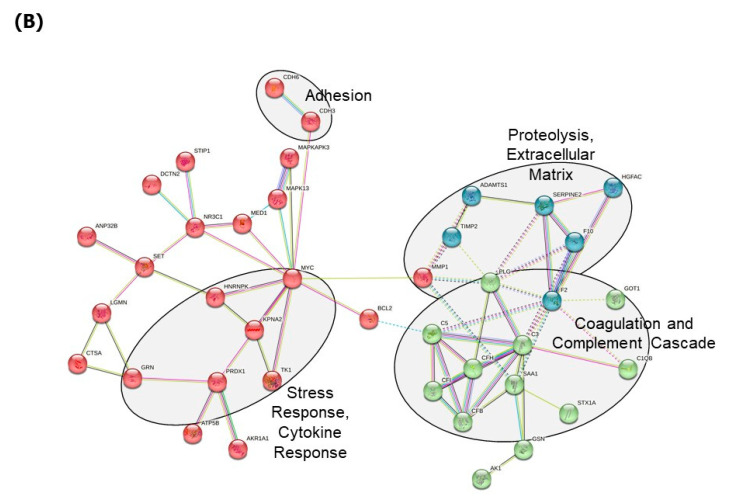
Systems biology analysis of 63 OSCC-associated proteins. (**A**) Biological functions that are significantly enriched by the input 63 protein list (**left** panel); upstream regulators that best explain the observed expression changes in OSCC (**right** panel); (**B**) Network and cluster analysis using the STRING database of functional and physical protein associations.

To identify the signaling pathways and biological mechanisms enriched significantly by the OSCC-associated proteins, we next performed an Ingenuity Pathway Analysis (IPA) using as the input the 63 OSCC-associated proteins. The IPA for bio functions converged on the enrichment of proteins associated with cell death (necrosis, necrosis of epithelial tissue, apoptosis, apoptosis of epithelial cells and cell death of epithelial cells), migration of immune cells (leukocyte migration, cell movement of leukocytes, cell movement of phagocytes, cell movement of neutrophils and cell movement of myeloid cells), anemia, proteolysis, neovascularization and cytolysis (Figure 2A). A total of 41 proteins were linked to a predicted decreased cell death of epithelial cells among the 63 OSCC-regulated proteins (for detail: Supplementary Figure S1A). Particularly informative was modeling the links between OSCC-associated proteins based on their established connections with predicted shared upstream regulatory proteins applying the Upstream Regulator Effect analysis. The highest statistically significant predicted upstream regulators converged on several pro-inflammatory cytokines (IL1A, IL6 and IL1B), cancer genes (HRAS, KRAS, MYC and BRCA1), transcription factors (CEBPB, SP1, KLF11, SMAD3, NFE2L2 and AHR) and extracellular matrix and fibrosis regulators (TGFB1 and TGFB3) as well as the androgen receptor (AR) and the microRNA miR-8 (red arrow in Figure 2A), indicating that these proteins (miRNA) may be involved in the dysregulation of a sizeable portion of the top 63 proteins in the serum signature for OSCC. Among these predicted upstream regulators, MYC, AR and miR-8 were especially potentially important master regulators of up- and down-regulated proteins in patients with OSCC (for detail: Supplementary Figure S1B).

Next, we performed a network and cluster analysis using the STRING database of functional and physical protein associations curated across the major data repositories. In total, 41 of the 63 proteins formed distinct interacting protein clusters that were enriched in pathways associated with adhesion, stress and cytokine response, proteolysis, extracellular matrix, complement cascade and coagulation (Figure 2B). The oncogene MYC occupied a key focus hub in this analysis linking all the above-listed biological functions together, and was itself reduced in the serum of OSCC patients compared with healthy controls (Table 2).

**Figure 3:**
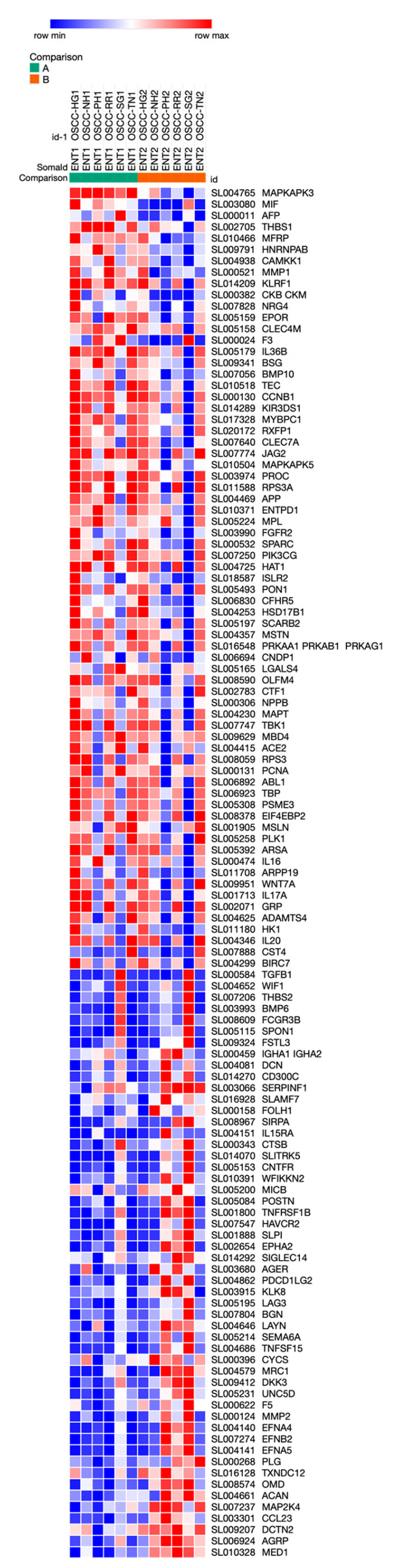
Heatmap of top 121 proteins discriminating between OSCC pre- (**left** column) and post-resection (**right** column, *p* < 0.05). Red = up-regulated, blue: down-regulated.

Furthermore, 121 proteins (49 increased, 72 decreased after resection) were detected that significantly differed between pre- and post-resection serum samples (Table 3, for details see Supplementary Table S2) and their differential expression is visualized by a heatmap in Figure 3. Here, the proteins that significantly increased post-resection included Ck-beta-8-1 (CCL23), osteomodulin (OMD), advanced glycosylation end product-specific receptor, soluble (AGER), dynactin-subunit 2 (DCTN2), biglycan (BGN), decorin (DCN) and Dickkopf-related protein 3 (DKK3), whereas proteins that decreased after resection included macrophage migration inhibitory factor (MIF), Tissue Factor (F3), thrombospondin-1 (THBS1) and interstitial collagenase (MMP1).

**Figure 4:**
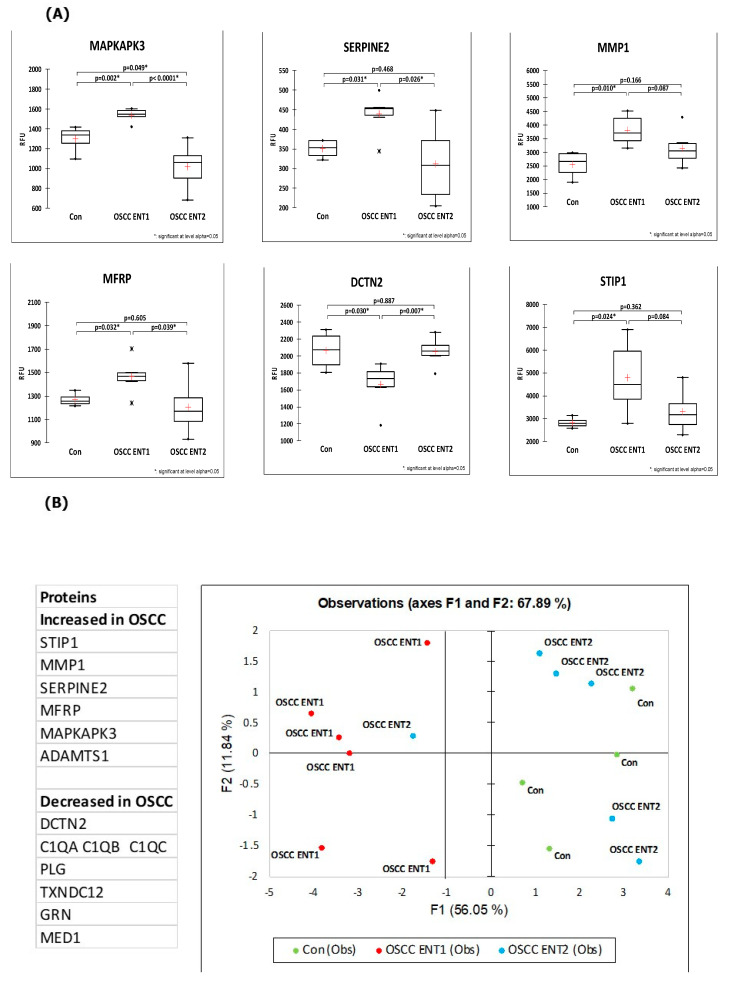
(**A**) The 12 OSCC-associated proteins reversed in expression after surgical resection. (**A**) Box Whisker plots for MAPKAPK2, SERPINE2, MMP1, MFRP, DCTN2 and STIP1 protein expression levels in pre-resection OSCC (OSCC ENT1), post-resection OSCC (OSCC ENT2) cases and healthy controls (Con). Data derived are shown as a Box Whisker plot with the mean expression depicted as + and median expression indicated by a horizontal line. The expression levels were determined by SOMAscan from patient serum. (**B**) PCA analysis using the 12 reversed OSCC-associated proteins. The names of all 12 proteins are shown.

Importantly, the expression levels of 12 out of the 63 OSCC-associated proteins reversed to levels equivalent to the healthy controls after resection, highlighting the potential relevance of these proteins as OSCC tumor markers (proteins are highlighted in bold in Table 2 and Table 3). Box Whisker plots show that the expression levels of five of these proteins increased in OSCC compared to the healthy controls but reduced upon resection, and one protein was decreased in OSCC and increased after resection (Figure 4A). To evaluate whether these 12 proteins (6 increased and6 decreased in OSCC compared to healthy controls) may represent a panel of biomarkers for discriminating OSCC from healthy controls and post-resection, we performed a PCA analysis. As shown in Figure 4B, the PCA resulted in the excellent separation of pre-resection OSCC cases (OSCC ENT1) from both the healthy controls and post-resection OSCC (OSCC ENT2), with only one OSCC case clustering post-resection with the pre-resection cases and the other post-resection OSCC cases clustering with the healthy controls.

**Figure 5:**
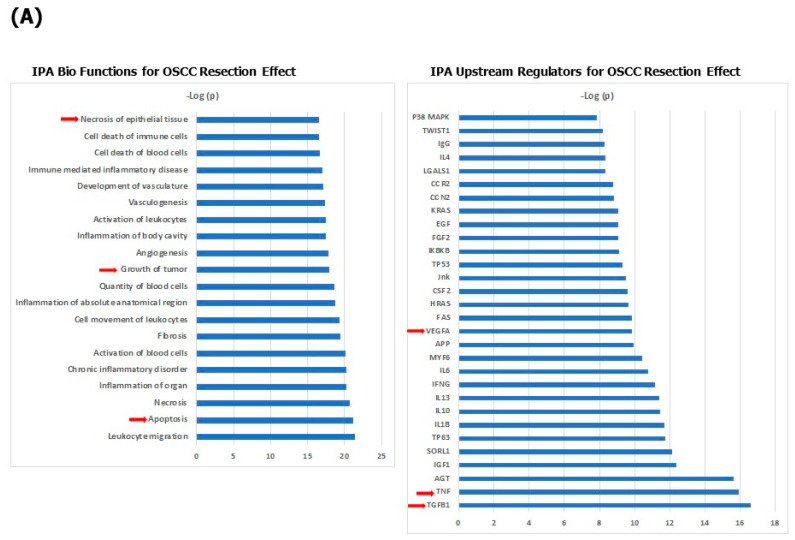

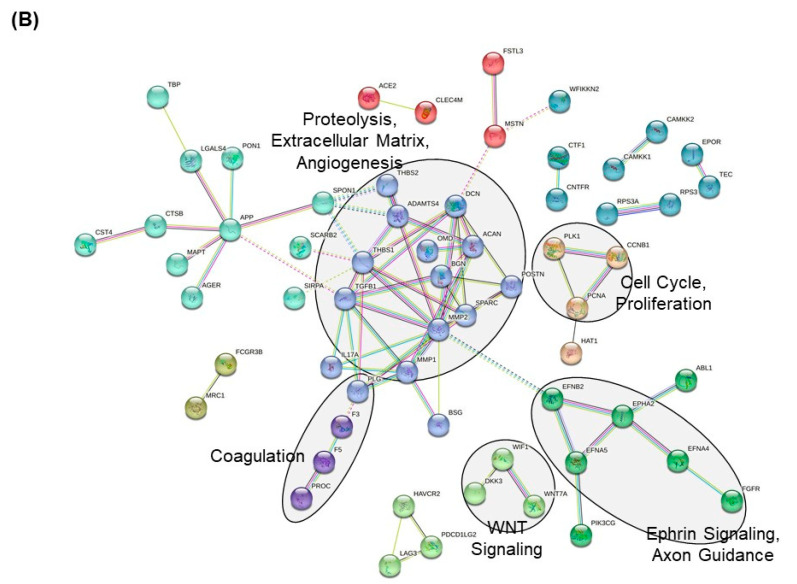
Systems biology analysis of 121 OSCC resection-associated proteins. (**A**) Biological functions that are significantly enriched by the input 121 protein list (**left** panel); Upstream regulators that best explain the observed expression changes in response to resection (**right** panel). (**B**) Network and cluster analysis using the STRING database of functional and physical protein associations.

A pathway analysis of the 121 proteins different between OSCCC pre- and post-resection showed various Bio Functions significantly enriched including an increase in apoptosis and necrosis of epithelial cells (Figure 5A and Figure S2A) as compared to decreased cell death in OSCC pre-resection as compared to healthy controls. Among the predicted enriched upstream regulators (Figure 5A), a reduced activity of TNF was predicted based on the regulation of 41 of the resection response proteins (Supplementary Figure S2B). Among the canonical pathways, the high-mobility group box 1 (HMGB1) protein signaling cascade, a key body–internal innate immune pathway was enriched to discriminate between pre- and post-resection sera (Supplementary Figure S2C). Last, a STRING analysis revealed distinct interacting protein clusters that were enriched in pathways associated with Wnt signaling, Ephrin signaling, axon guidance, cell cycle, proliferation, proteolysis, angiogenesis, extracellular matrix and coagulation (Figure 5B).

## 4. Discussion

This exploratory study is the first to evaluate the sera of patients with oral squamous cell carcinoma (OSCC) vs. healthy controls and pre- vs. post-resection using a highly multiplexed and specific proteomic technology employing protein-specific DNA-aptamers (SOMAscan^®^ technology) that captures 1305 proteins which may qualify as potential OSCC liquid biomarkers. As the major finding of this analysis, the approach seems feasible and significantly distinguishes between the disease and healthy controls in this specific type of cancer. Furthermore, the analysis of pre- and post-resection sera shows statistically significant differences in serum protein abundances that may help to monitor possible tumor recurrence, and even the early response to therapy. Most interestingly, the expression of several OSCC-associated proteins reverted to healthy control levels after resection. Lastly, pathway and interactive network analyses shed a light on the herein involved molecular pathways and may reveal novel protein biomarkers that were not detectable with prior proteomic platforms. The key advantage of SOMAscan proteomics is the unprecedented dynamic range down to low pg/mL levels that allows to interrogate serum proteins across the whole dynamic range.

There is rising evidence that analyzing the proteome may reveal a prognostic signature of OSCC and help to optimize diagnostics and individualize therapy concepts [31]. This way, multiple protein biomarker events that can serve as molecular fingerprints for the assessment of disease state and prognosis may be detected [18,32]. However, such earlier studies were limited due to the difficulty to detect and especially quantify thousands of serum or plasma proteins of low abundancy that are usually the most specific markers of disease, but reflect less than 1% of the total protein content dominated by abundant proteins such as albumins, immunoglobulins, common transport proteins and others. Thus, the discovery of suitable disease biomarkers has long been hampered by the complexity and a huge dynamic range of serum/plasma protein concentrations across more than 12 logs combined with the lack of proteomic technologies to measure large numbers of proteins across such a broad dynamic range. Recent achievements in technologies addressed these limitations to specify and quantify the human plasma and serum proteome and improve biomarker development [17,20,23]. Among these, 2-gel electrophoresis (2DE) coupled with matrix-assisted laser desorption time-of-flight mass spectrometry (MALDI-TOF), as well as two-dimension liquid chromatography in combination with tandem mass spectrometry, were considered as state-of-the-art for almost a decade in discovery proteomics. However, the application of affinity-based, high multiplex methods such as the here-used aptamer-based SOMAscan, or the proximity extension assay (PEA, Olink), technologies are being applied increasingly with great success to an ever increasing number of clinically relevant studies [17,18,20,21,22,23,26,33]. So far, the SOMAscan technology has been linked to the biomarker discovery of various chronic and acute diseases [22,24,25,34,35,36,37,38,39,40]. For cancer, the assay is prescribed feasible for hepatocellular carcinoma [22], ovarian cancer [41,42], non-small lung cancer [43] and malignant glioma [44,45].

Notably, an aptamer-based technology can show a satisfying within-person stability of archived samples for up to 91% of proteins over 1 year [46]. However, the disadvantages of the technique have been discussed to include a missing correlation between some target proteins and the SOMAmers used for capture and detection, as well as cross-reactivity with and comparable binding to highly homologous but different proteins, likely due to lack of specificity for some aptamers or antibodies (that are used in the PEA technology), or due to differences in affinity and thus signal-to-noise ratios [47]. Moreover, there usually exist various isoforms for each protein, degradation products, posttranslational modifications and protein–protein interaction-based complex formations, and some aptamers (or antibodies) may recognize a particular proteoform that is different from the one recognized in an immunoassay.

Within this exploratory study, a wide range of identical but also different proteins that were differentially expressed between controls and OSCC and between pre- and post-resection samples were found in every investigated hierarchical level. This may be seen as a reflection of the known tumor heterogeneity of oral carcinoma, as of other cancers, that complicates biomarker development. Thus, a recent study using a combined discovery and targeted proteomics approach in (1) formalin-fixed paraffin-embedded (FFPE) OSCC tissues and (2) saliva samples of oral cancer patients suggested that tumors are able to differentially express proteome profiles according to their spatial distribution in the tumor center, micromilieu, neoplastic islands and stroma [31].

Although further elucidating the specific role of the detected proteins, e.g., by extension of the SOMAscan analysis to a larger series of well-defined patients with OSCC is beyond the aim of this study, 63 proteins were able to discriminate accurately between the healthy controls and the tumor patients. As important representatives, tissue inhibitor of matrix metalloproteinases-2 (TIMP2), interstitial collagenase MMP-1, gelsolin, peroxiredoxin 1 and glia-derived nexin were significantly up-regulated, and various components of the complement cascade, cathepsin A and angiostatin were down-regulated in the tumor vs. healthy control group. Notably, several of these proteins were previously found to be up- or down-regulated in tissue specimens or cell cultures of OSCC vs. controls [48,49,50,51,52,53,54].

The additional pathway analysis based on the regulated serum proteome also suggested that the reduced cell death of epithelial cells appears to play an important role in OSCC. An up-stream regulator analysis predicted MYC and androgen receptor (AR) to regulate various proteins from the OSCC protein signature and may reveal further, here undetected, potential serum biomarkers for assay development. The androgen receptor is of significant interest for OSCC, as the overexpression of AR in OSCC has been linked as a predictor of disease progression [55]. The Myc pathway is implicated in proto-oncogenesis driving cell proliferation, differentiation and the epithelial–mesenchymal transition in (oral) carcinogenesis [56] and a STRING analysis of protein–protein interactions places Myc as a focus hub linking it to various biological functions.

Additionally, several serum proteins were detected that could distinguish with high sensitivity between pre- and post-resection patients, partly overlapping with proteins differentiating patients with OSCC from the healthy controls. The expression levels of 12 of these 63 OSCC-associated protein biomarkers reverted to levels seen in healthy controls after surgical tumor resection, demonstrating that at least a subset of these proteins may be plausible causal proteins expressed in the tumor cells themselves. Among these 12 proteins, several were overexpressed proteins, with links to OSCC or cancer such as interstitial collagenase (MMP1), stress-induced-phosphoprotein 1 (STIP1), glia-derived nexin (SERPINE2), membrane frizzled-related protein (MFRP) and MAP kinase-activated protein kinase 3 (MAPKAPK3). For example, STIP1 is overexpressed in OSCC and its expression directly correlates with poor survival and an increased risk of recurrence [57]. SERPINE2 has also been reported to be highly expressed in OSCC and to induce angiogenesis [58]. Interestingly, a PCA using these 12 OSCC-associated proteins reversed by resection clustered the post-resection OSCC cases with the healthy controls, demonstrating the reversal of the OSCC phenotype and indicating the potential to use this 12-protein panel for monitoring tumor recurrence.

In addition, the pathway analysis revealed high-mobility group box 1 (HMGB1) signaling as important to discriminate between pre- and post-resection sera. This is in accordance with other studies [59], were HMGB1 was suggested as a possible key promoter of tumor progression and infiltration of the mandible bone or the upper jaw in OSCC [60]. Furthermore, HMGB1 serum levels had previously been evaluated as a possible marker to predict a favorable outcome during the adjuvant radio-chemotherapy of patients with OSCC [61].

A limitation of our study is its exploratory nature, with a small sample size of patients and controls, and a lack of patients who developed recurrence or metastasis. However, patients were very well characterized, a precondition for a reliable interpretation of high-end proteomics. Due to the small sample size, a possible association between the age and sex of the patients and the discovered biomarkers was not analyzed. Furthermore, no severe comorbidities of the patients were recorded but it cannot be ruled out that the well characterized, moderate co-morbidities did influence the presented results. Additionally, no severe trauma or inflammation was included in the control group, but the influence of implant surgery and extraction of destroyed but not infected teeth on the discovered biomarkers was beyond the scope of this study. Despite the limited number of sera, all analyses, especially the hierarchical cluster analysis, revealed significant differences between 63 proteins of the tumor sera and the sera of healthy controls, many of them with high biological plausibility. Although statistical testing and demonstrated significances should be interpreted with caution due to the low sample size and possible type 2 errors, our pilot study shows feasibility and sets the stage for diagnostic and monitoring serum assay development and especially subsequent broader scale studies in this field, as generally recommended in the literature [62]. Follow-up studies, either via extended high-end proteomics and accompanying ELISA development and validation, should benefit OSCC patients by better predicting the response to radio or chemotherapy, lymph node metastasis, tumor recurrence and overall survival.

## 5. Conclusions

In summary, the presented findings support the hypothesis that high-multiplex quantitative serum proteome analysis will lead to useful novel serum/plasma markers for the early diagnosis, prognosis, recurrence and optimal therapeutic management of patients with OSCC.

## Figures and Tables

**Table 1:** Characterization of OSCC and age-matched healthy control patients.

Patient	Gender	Age	Comorbidity	Tumor Site	Status			Grading	Adjuvant Therapy
					T	N	M		
1	Male	70	Coronary heart disease	Floor of the mouth	4	0	0	2	Irradiation (64 Gray)
2	Female	63	None	Cheek	2	1	0	2	Irradiation (64 Gray)
3	Male	62	None	Floor of the mouth	4	0	0	2	Irradiation (64 Gray)
4	Male	80	Chronic venous disease	Hard palate	2	0	0	2	none
5	Male	68	Arterial hypertension	Floor of the mouth	4	2	0	3	Irradiation (64 Gray)
6	Male	63	None	Soft palate	1	0	0	2	none
7	Male	63	Arterial hypertension	-	-	-	-	-	-
8	Male	75	Depression	-	-	-	-	-	-
9	Female	68	None	-	-	-	-	-	-
10	Female	85	Arterial hypertension, Coronary heart disease	-	-	-	-	-	-

**Table 2:** Top Up-Regulated and Down-Regulated Proteins Between OSCC Cases and Healthy Controls.

Down-regulated Proteins between OSCC and Healthy Control	Up-regulated Proteins between OSCC and Healthy Control
Adenylate kinase isoenzyme 1	Heterogeneous nuclear ribonucleoprotein K
Hemoglobin	Complement C5
Xaa-Pro aminopeptidase 1	Complement factor I
Alcohol dehydrogenase [NADP(+)]	Complement factor H
Casein kinase II 2-alpha:2-beta heterotetramer	Complement component 1 Q subcomponent-binding protein, mitochondrial
**Stress-induced-phosphoprotein 1**	Syntaxin-1A
Peroxiredoxin-1	Myc proto-oncogene protein
Interleukin-3 receptor subunit alpha	Serine protease 27
**Interstitial collagenase**	Mammaglobin-B
Desert hedgehog protein N-product	Coagulation Factor X
Glucocorticoid receptor	Thymidine kinase, cytosolic
Metalloproteinase inhibitor 2	**Dynactin subunit 2**
**Glia-derived nexin**	Lymphocyte antigen 86
Kallikrein-8	Peroxisomal targeting signal 1 receptor
Gelsolin	Legumain
Importin subunit alpha-1	Ubiquitin-conjugating enzyme E2 G2
**Membrane frizzled-related protein**	C3a anaphylatoxin des Arginine
Apoptosis regulator Bcl-2	**Complement C1q subcomponent**
**MAP kinase-activated protein kinase 3**	Cadherin-3
A disintegrin and metalloproteinase with thrombospondin motifs 1	Parathyroid hormone-related protein
	Cadherin-6
Kremen protein 2	HERV-H LTR-associating protein 2
	Semaphorin-3A
	Mitogen-activated protein kinase 13
	Complement factor B
	**Angiostatin**
	Growth/differentiation factor 9
	**Thrombin**
	Aspartate aminotransferase, cytoplasmic
	Granulocyte colony-stimulating factor receptor
	Endothelin-converting enzyme 1
	**Granulins**
	**Mediator of RNA polymerase II transcription** **subunit 1**
	Lysosomal protective protein
	SLAM family member 6
	**Protein SET**
	Interleukin-1 Receptor accessory protein
	Hepatocyte growth factor activator
	Acidic leucine-rich nuclear phosphoprotein 32 family member B
	ATP synthase subunit beta, mitochondrial
	E3 SUMO-protein ligase PIAS4
	Serum amyloid A-1 protein

**Table 3:** Top Up-Regulated and Down-Regulated Proteins Between ENT2 and ENT1 in OSCC Cases.

Increased in ENT2 in OSCC	Decreased in ENT2 in OSCC
**Ck-beta-8-1**	Creatine kinase M-type:Creatine kinase B-type heterodimer
Osteomodulin	Macrophage migration inhibitory factor
Macrophage mannose receptor 1	Tissue Factor
Ephrin type-A receptor 2	Beta-Ala-His dipeptidase
Lymphocyte activation gene 3 protein	SPARC
SLIT and NTRK-like protein 5	Thrombospondin-1
Dual specificity mitogen-activated protein kinase kinase 4	**MAP kinase-activated protein kinase 3**
Hepatitis A virus cellular receptor 2	Complement factor H-related protein 5
Netrin receptor UNC5D	Myosin-binding protein C, slow-type
Ephrin-B2	**Membrane frizzled-related protein**
Advanced glycosylation end product-specific receptor, soluble	Estradiol 17-beta-dehydrogenase 1
	alpha-Fetoprotein
Tumor necrosis factor ligand superfamily member 15	**Interstitial collagenase**
Ephrin-A5	Tyrosine-protein kinase Tec
72 kDa type IV collagenase	Calcium/calmodulin-dependent protein kinase kinase 1
Tumor necrosis factor receptor superfamily member 1B	Hexokinase-1
Antileukoproteinase	**Heterogeneous nuclear ribonucleoprotein A/B**
Spondin-1	Basigin
Follistatin-related protein 3	Amyloid beta A4 protein
Dickkopf-related protein 3	C-type lectin domain family 7 member A
Agouti-related protein	Erythropoietin receptor
**Mediator of RNA polymerase II transcription subunit 1**	Thrombopoietin Receptor
Semaphorin-6A	Bone morphogenetic protein 10
Ephrin-A4	Activated Protein C
Ciliary neurotrophic factor receptor subunit alpha	Serine/threonine-protein kinase TBK1
Periostin	Angiotensin-converting enzyme 2
Thrombospondin-2	Relaxin receptor 1
Cytochrome c	Phosphatidylinositol 4,5-bisphosphate 3-kinase catalytic subunit gamma isoform
Glutamate carboxypeptidase 2	
Aggrecan core protein	Cystatin-S
**Programmed cell death 1 ligand 2**	Mesothelin
Low affinity immunoglobulin gamma Fc region receptor III-B	Immunoglobulin superfamily containing leucine-rich repeat protein 2
Biglycan	Serine/threonine-protein kinase PLK1
Kallikrein-8	40S ribosomal protein S3a
WAP, Kazal, immunoglobulin, Kunitz and NTR domain-containing protein 2	Protein Wnt-7a
	Brain natriuretic peptide 32
Decorin	Eukaryotic translation initiation factor 4E-binding protein 2
CMRF35-like molecule 6	cAMP-regulated phosphoprotein 19
Coagulation Factor V	40S ribosomal protein S3
SLAM family member 7	Killer cell lectin-like receptor subfamily F member 1
**Thioredoxin domain-containing protein 12**	Microtubule-associated protein tau
Tyrosine-protein phosphatase non-receptor type substrate 1	Killer cell immunoglobulin-like receptor 3DS1
	Histone acetyltransferase type B catalytic subunit
**Angiostatin**	Gastrin-releasing peptide
**Dynactin subunit 2**	Interleukin-36 beta
Bone morphogenetic protein 6	G2/mitotic-specific cyclin-B1
Cathepsin B	Protein jagged-2
Layilin	AMP Kinase (alpha1beta1gamma1)
MHC class I polypeptide-related sequence B	Proteasome activator complex subunit 3
Pigment epithelium-derived factor	Serum paraoxonase/arylesterase 1
Interleukin-15 receptor subunit alpha	Growth/differentiation factor 8
Sialic acid-binding Ig-like lectin 14	Olfactomedin-4
Wnt inhibitory factor 1	Ectonucleoside triphosphate diphosphohydrolase 1
Immunoglobulin A	MAP kinase-activated protein kinase 5
	Arylsulfatase A
	C-type lectin domain family 4 member M
	Tyrosine-protein kinase ABL1
	Lysosome membrane protein 2
	A disintegrin and metalloproteinase with thrombospondin motifs 4
	Methyl-CpG-binding domain protein 4
	Cardiotrophin-1
	Proliferating cell nuclear antigen
	Baculoviral IAP repeat-containing protein 7 Isoform beta
	Neuregulin-4
	Transforming growth factor beta-1
	Interleukin-20
	Fibroblast growth factor receptor 2
	TATA-box-binding protein
	Interleukin-16
	Interleukin-17A
	Galectin-4

## Data Availability

All data are shown in the study and in the Supplementary Material.

## Supplementary Materials

The following supporting information can be downloaded at: https://www.mdpi.com/article/10.3390/cancers15072071/s1. Figure S1A,B: Systems biology analysis of 63 OSCC-associated proteins. The OSCC-associated proteins linked to cell death of epithelial cells, apoptosis, and necrosis. Red = upregulation, Green = downregulation of the targets. Arow: directions of impact. Systems biology analysis of 63 OSCC-associated proteins. MYC, AR, and miR-8 interactions as upstream regulators. Red = upregulation, Green = downregulation of the targets. Arow: directions of impact, T-sign: in-hibition. Orange lines represent activated states and blue lines represent inhibited states. Yellow represents indeterminate and grey if the experimental findings do not agree with the predicted effect. Red for the biological effects indicates enhanced activity and blue reduced activity. Figure S2A–C: Systems biology analysis of 121 OSCC resection-associated proteins. The OSCC resec-tion-associated proteins linked to necrosis of epithelial cells, apoptosis, and necrosis. Systems biology analysis of 121 OSCC resection-associated proteins. TNF interactions as upstream regulators. Red = upregulation, Green = downregulation of the targets. Arrow: directions of impact, T-sign: inhibition. Orange lines represent activated states and blue lines represent inhibited states. Yellow represents indeterminate and black if the experimental findings do not agree with the predicted effect. Red for the biological effects indicates enhanced activity and blue reduced activity. Systems biology analysis of 121 OSCC resection-associated proteins. High-mobility group box 1 (HMGB1) pathway as key regulator to discriminate between pre- and post-resection sera (p < 0.05). Red = upregulated, green = downregulated. Table S1: Top Up-Regulated and Down-Regulated Proteins Between OSCC Cases and Healthy Controls. Table S2: Top Up-Regulated and Down-Regulated Proteins Between ENT2 and ENT1 in OSCC Cases.
