# Supplementary material for: High-Multiplex Aptamer-Based Serum Proteomics to Identify Candidate Serum Biomarkers of Oral Squamous Cell Carcinoma
Source: Cancers (Basel). 2023 Mar 30;15(7):2071. doi: 10.3390/cancers15072071 (PMC10093013; doi:10.3390/cancers15072071)
Supplement: Supplementary file 1 [file cancers-15-02071-s001.zip › cancers-2276052-supplementary.pdf]

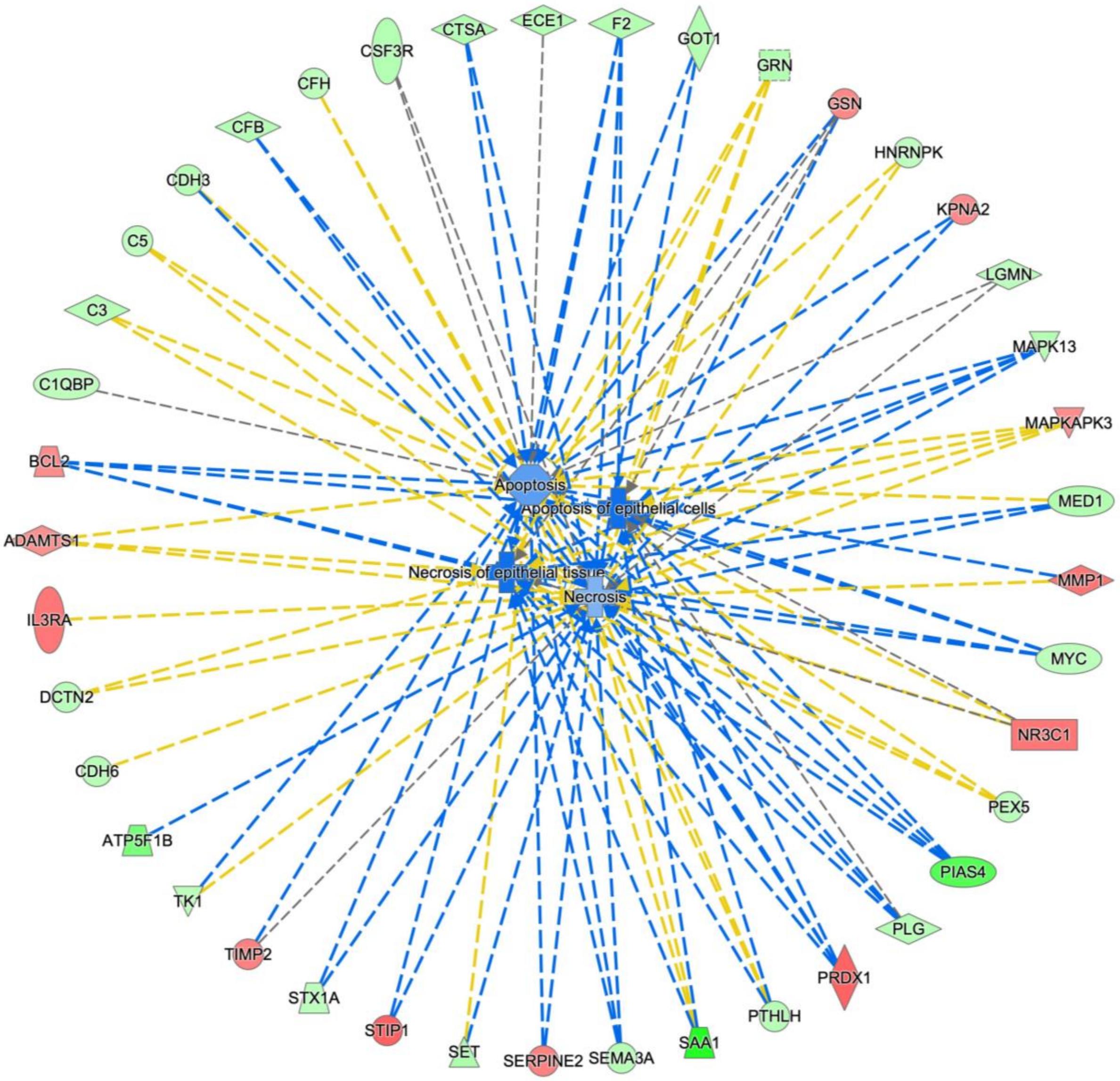

Supplementary Figure S1A. Systems biology analysis of 63 OSCC-associated proteins. The OSCC-associated proteins linked to cell death of epithelial cells, apoptosis, and necrosis. Red = upregulation, Green = downregulation of the targets. Arow: directions of impact.

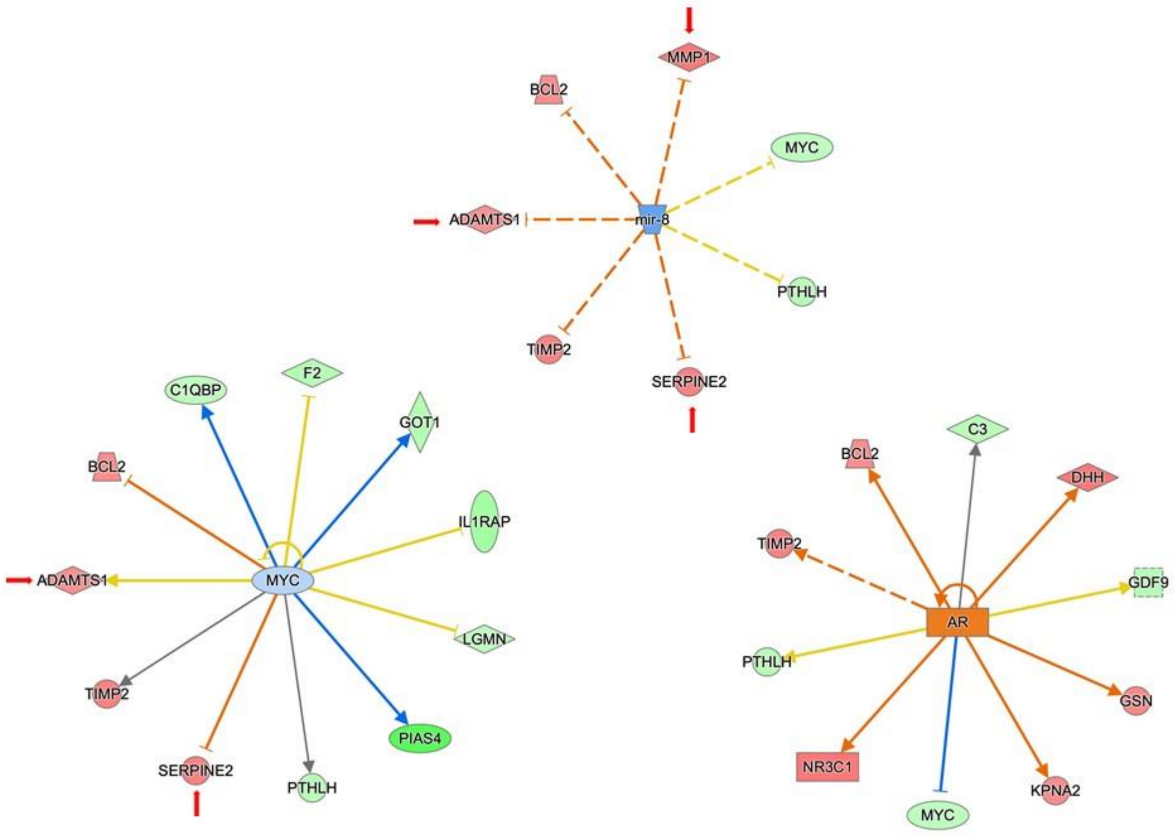

Supplementary Figure S1B. Systems biology analysis of 63 OSCC-associated proteins. MYC, AR, and miR-8 interactions as upstream regulators. Red = upregulation, Green = downregulation of the targets. Arow: directions of impact, T-sign: inhibition. Orange lines represent activated states and blue lines represent inhibited states. Yellow represents indeterminate and grey if the experimental findings do not agree with the predicted effect. Red for the biological effects indicates enhanced activity and blue reduced activity.

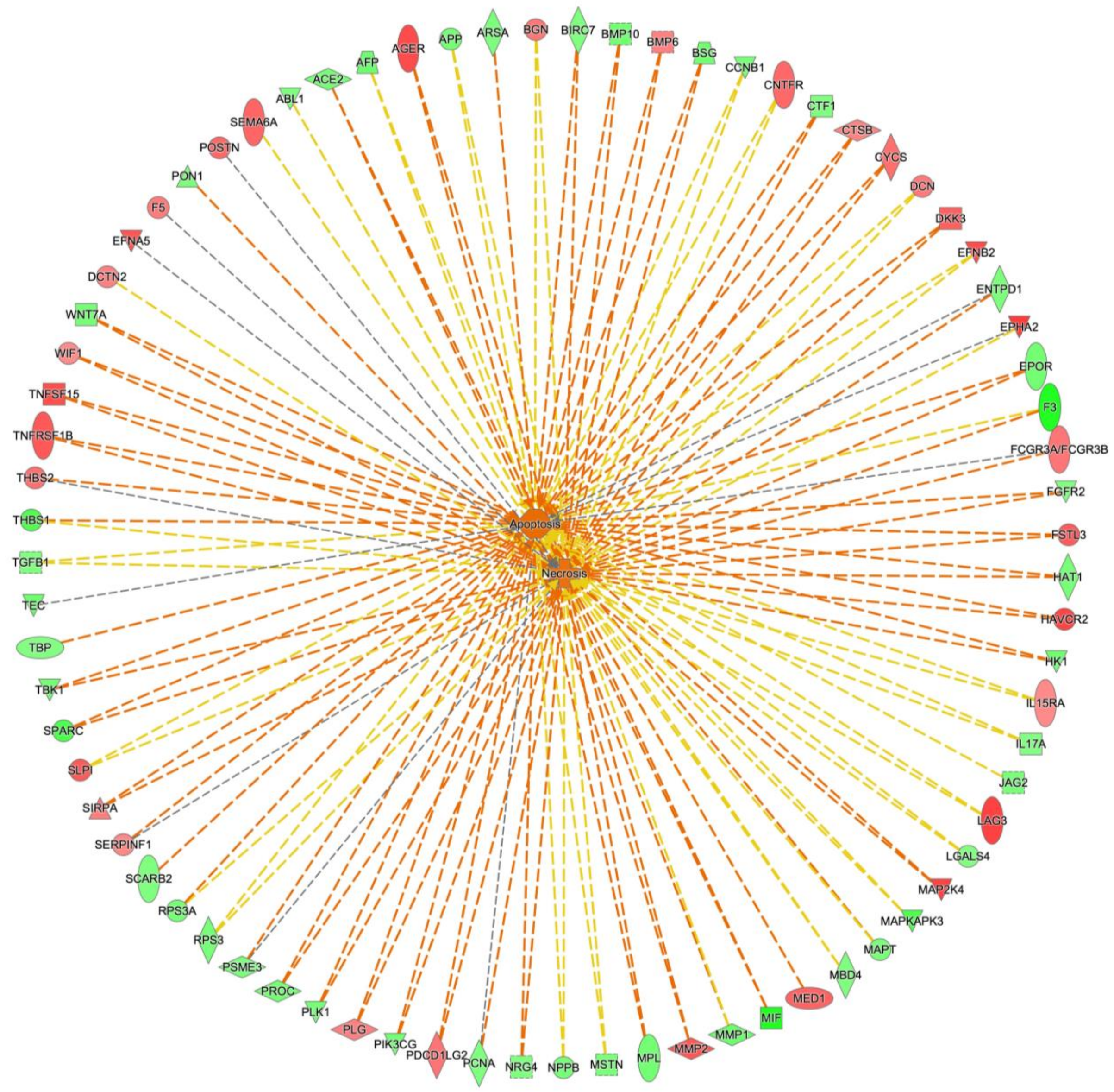

Supplementary Figure S2A. Systems biology analysis of 121 OSCC resection-associated proteins. The OSCC resection-associated proteins linked to necrosis of epithelial cells, apoptosis, and necrosis.

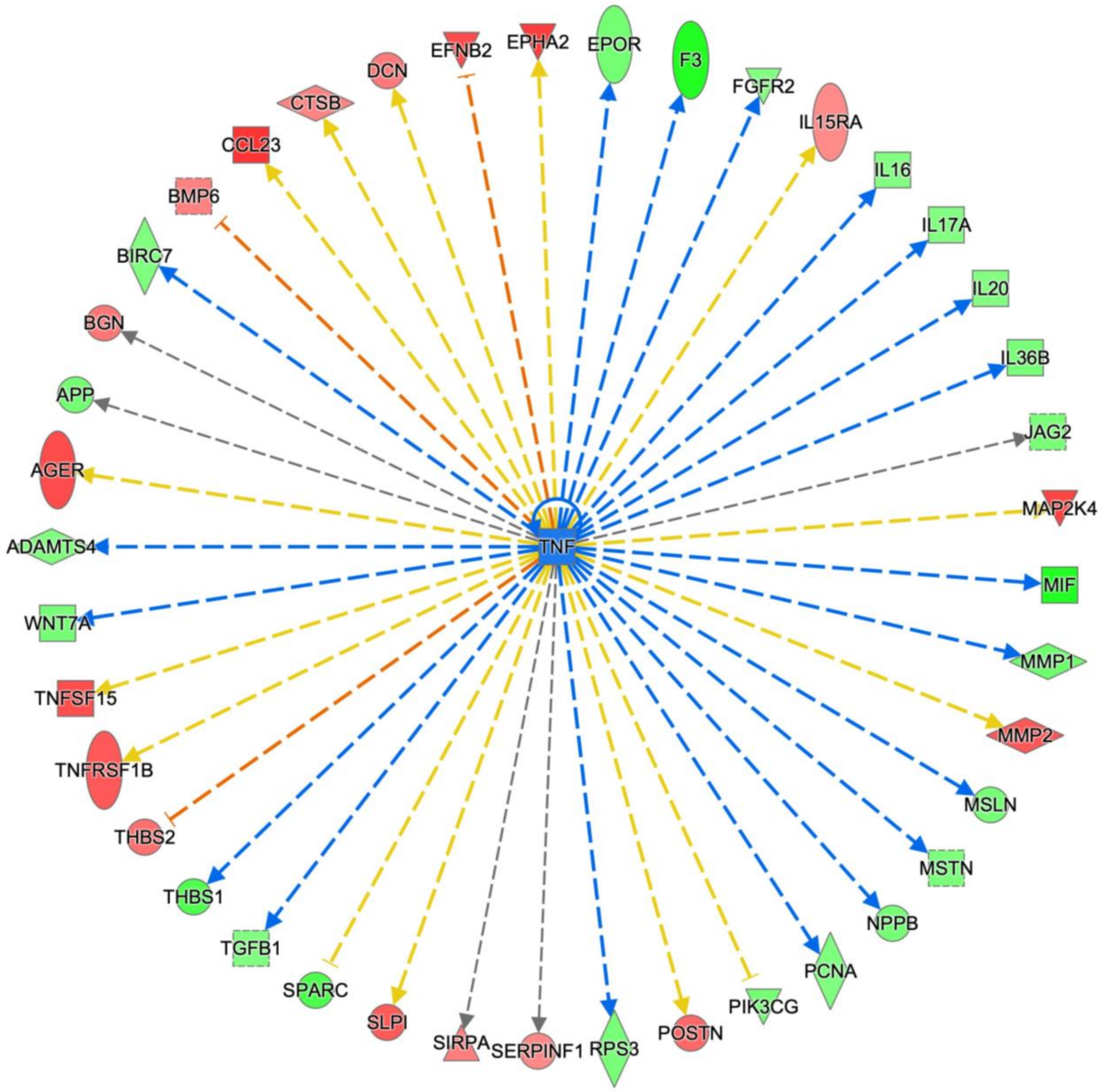

Supplementary Figure S2B. Systems biology analysis of 121 OSCC resection-associated proteins. TNF interactions as upstream regulators. Red = upregulation, Green = downregulation of the targets. Arrow: directions of impact, T-sign: inhibition. Orange lines represent activated states and blue lines represent inhibited states. Yellow represents indeterminate and black if the experimental findings do not agree with the predicted effect. Red for the biological effects indicates enhanced activity and blue reduced activity.

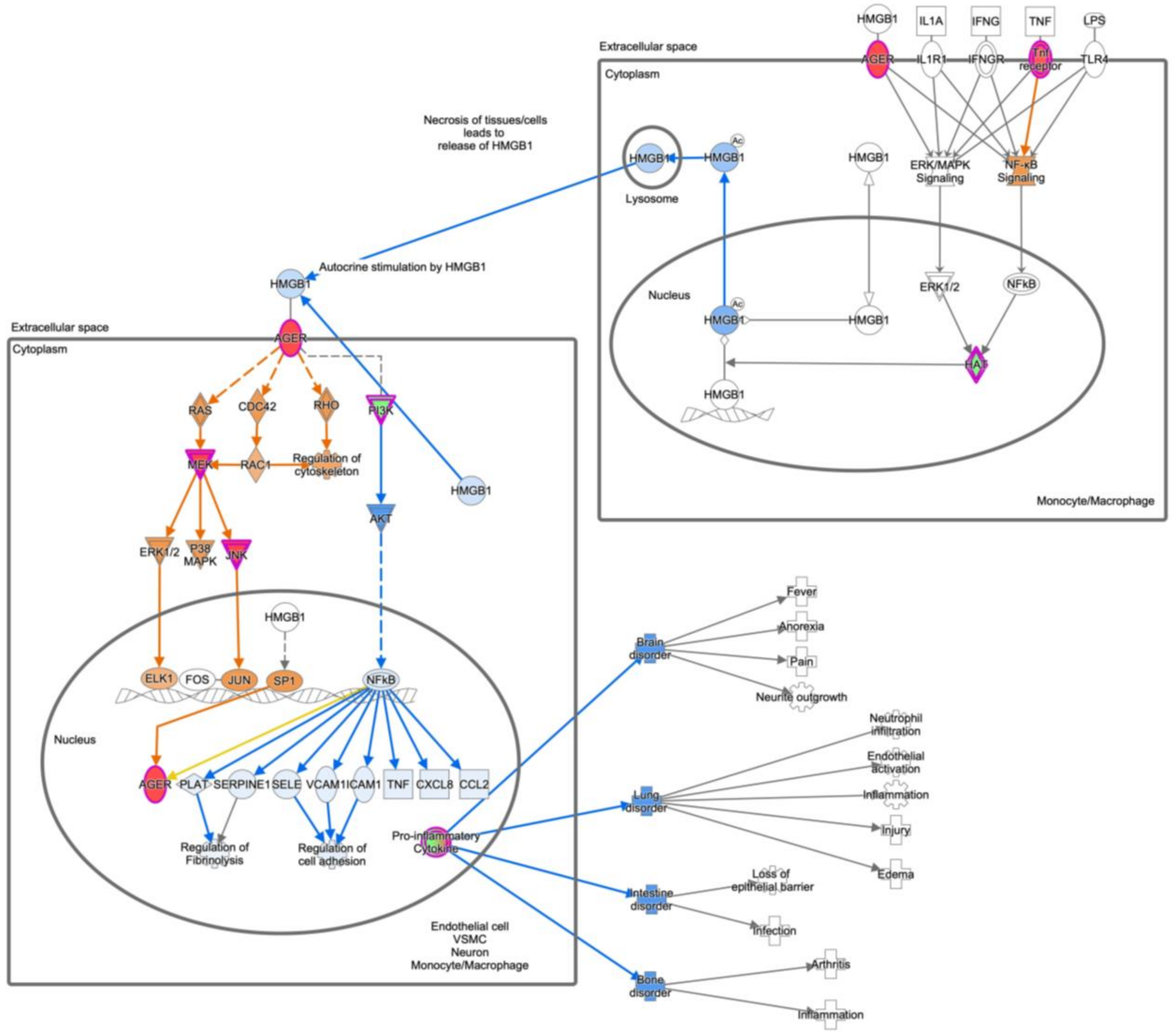

Supplementary Figure S2C. Systems biology analysis of 121 OSCC resection-associated proteins. High-mobility group box 1 (HMGB1) pathway as key regulator to discriminate between pre- and post-resection sera (p<0.05). Red = upregulated, green = downregulated.

Supplementary Table S1. Top Up-Regulated and Down-Regulated Proteins Between ENT2 and ENT1 in OSCC Cases

Increased in ENT2 in OSCC

					OSCC ENT2 vs ENT1		
**SomaId**	**Protein Name**	**Target**	**Entrez Gene Symbol**		**p-val**	**mean FC**	**median FC**
**SL003301**	**Ck-beta-8-1**	**Ck-b-8-1**	**CCL23**		**0.03302463**	**1.81**	**1.93**
SL008574	Osteomodulin	OMD	OMD		0.03472337	1.69	1.90
SL004579	Macrophage mannose receptor 1	Macrophage mannose receptor	MRC1		0.04120763	1.44	1.89
SL002654	Ephrin type-A receptor 2	Epithelial cell kinase	EPHA2		0.03671686	1.59	1.83
SL005195	Lymphocyte activation gene 3 protein	LAG-3	LAG3		0.03762533	1.71	1.78
SL014070	SLIT and NTRK-like protein 5	SLIK5	SLITRK5		0.04633227	1.60	1.77
SL007237	Dual specificity mitogen-activated protein kinase kinase 4	MP2K4	MAP2K4		0.01697816	1.67	1.76
SL007547	Hepatitis A virus cellular receptor 2	TIMD3	HAVCR2		0.03365328	1.71	1.75
SL005231	Netrin receptor UNC5D	UNC5H4	UNC5D		0.03117104	1.58	1.70
SL007274	Ephrin-B2	EFNB2	EFNB2		0.03265511	1.76	1.69
SL003680	Advanced glycosylation end product-specific receptor, soluble	sRAGE	AGER		0.02730538	1.64	1.68
SL004686	Tumor necrosis factor ligand superfamily member 15	TNFSF15	TNFSF15		0.02785529	1.72	1.66
SL004141	Ephrin-A5	Ephrin-A5	EFNA5		0.03781306	1.57	1.61
SL000124	72 kDa type IV collagenase	MMP-2	MMP2		0.04128451	1.48	1.57
SL001800	Tumor necrosis factor receptor superfamily member 1B	TNF sR-II	TNFRSF1B		0.04152369	1.38	1.57
SL001888	Antileukoproteinase	SLPI	SLPI		0.03650918	1.41	1.55
SL005115	Spondin-1	Spondin-1	SPON1		0.0475309	1.27	1.53
SL009324	Follistatin-related protein 3	FSTL3	FSTL3		0.042269	1.33	1.53
SL009412	Dickkopf-related protein 3	DKK3	DKK3		0.01982	1.46	1.50
SL006924	Agouti-related protein	ART	AGRP		0.02632341	1.39	1.49
**SL010328**	**Mediator of RNA polymerase II transcription subunit 1**	**MED-1**	**MED1**		**0.00419701**	**1.38**	**1.46**
SL005214	Semaphorin-6A	Semaphorin-6A	SEMA6A		0.02577428	1.62	1.44
SL004140	Ephrin-A4	Ephrin-A4	EFNA4		0.04090624	1.37	1.43
SL005153	Ciliary neurotrophic factor receptor subunit alpha	CNTFR alpha	CNTFR		0.03959794	1.53	1.42
SL005084	Periostin	Periostin	POSTN		0.03877483	1.38	1.42
SL007206	Thrombospondin-2	TSP2	THBS2		0.04459738	1.25	1.35
SL000396	Cytochrome c	Cytochrome c	CYCS		0.03612491	1.27	1.35
SL000158	Glutamate carboxypeptidase 2	PSMA	FOLH1		0.04540058	1.33	1.35
SL004661	Aggrecan core protein	Aggrecan	ACAN		0.03675978	1.31	1.31
**SL004862**	**Programmed cell death 1 ligand 2**	**PD-L2**	**PDCD1LG2**		**0.04814705**	**1.41**	**1.31**
SL008609	Low affinity immunoglobulin gamma Fc region receptor III-B	FCG3B	FCGR3B		0.03538217	1.22	1.29
SL007804	Biglycan	BGN	BGN		0.01890219	1.45	1.29
SL003915	Kallikrein-8	kallikrein 8	KLK8		0.0498659	1.28	1.28
SL010391	WAP, Kazal, immunoglobulin, Kunitz and NTR domain-containing protein 2	WFKN2	WFIKKN2		0.045237	1.55	1.28
SL004081	Decorin	Bone proteoglycan II	DCN		0.02037425	1.23	1.26
SL014270	CMRF35-like molecule 6	CLM6	CD300C		0.04696386	1.37	1.25
SL000622	Coagulation Factor V	Coagulation Factor V	F5		0.03328398	1.23	1.24
SL016928	SLAM family member 7	SLAF7	SLAMF7		0.03934254	1.32	1.23
**SL016128**	**Thioredoxin domain-containing protein 12**	**TXD12**	**TXNDC12**		**0.0303045**	**1.17**	**1.21**
SL008967	Tyrosine-protein phosphatase non-receptor type substrate 1	SHPS1	SIRPA		0.04851118	1.34	1.21
**SL000268**	**Angiostatin**	**Angiostatin**	**PLG**		**0.04848184**	**1.21**	**1.19**
**SL009207**	**Dynactin subunit 2**	**Dynactin subunit 2**	**DCTN2**		**0.01861232**	**1.23**	**1.19**
SL003993	Bone morphogenetic protein 6	BMP-6	BMP6		0.04111271	1.17	1.19
SL000343	Cathepsin B	Cathepsin B	CTSB		0.01261039	1.15	1.16
SL004646	Layilin	Layilin	LAYN		0.03565695	1.11	1.12
SL005200	MHC class I polypeptide-related sequence B	MICB	MICB		0.02680499	1.21	1.12
SL003066	Pigment epithelium-derived factor	PEDF	SERPINF1		0.0199362	1.09	1.12
SL004151	Interleukin-15 receptor subunit alpha	IL-15 Ra	IL15RA		0.01978671	1.13	1.10
SL014292	Sialic acid-binding Ig-like lectin 14	SIG14	SIGLEC14		0.0307794	1.27	1.09
SL004652	Wnt inhibitory factor 1	WIF-1	WIF1		0.00808169	1.07	1.07
SL000459	Immunoglobulin A	IgA	IGHA1 IGHA2		0.04454073	1.13	1.04

**Decreased in ENT2 in OSCC**							
					**OSCC ENT2 vs ENT1**		
**SomaId**	**Protein Name**	**Target**	**Entrez Gene Symbol**		**p-val**	**mean FC**	**median FC**
SL000382	Creatine kinase M-type:Creatine kinase B-type heterodimer	CK-MB	CKB CKM		0.02986242	-1.55	-1.93
SL003080	Macrophage migration inhibitory factor	MIF	MIF		0.03432143	-1.66	-1.86
SL000024	Tissue Factor	TF	F3		0.04665021	-1.54	-1.83
SL006694	Beta-Ala-His dipeptidase	CNDP1	CNDP1		0.03254756	-1.32	-1.55
SL000532	SPARC	ON	SPARC		0.0266873	-1.32	-1.47
SL002705	Thrombospondin-1	Thrombospondin-1	THBS1		0.00051756	-1.42	-1.46
**SL004765**	**MAP kinase-activated protein kinase 3**	**MAPKAPK3**	**MAPKAPK3**		**0.00123178**	**-1.51**	**-1.46**
SL006830	Complement factor H-related protein 5	complement factor H-related 5	CFHR5		0.0168557	-1.29	-1.35
SL017328	Myosin-binding protein C, slow-type	MYPC1	MYBPC1		0.00678106	-1.24	-1.27
**SL010466**	**Membrane frizzled-related protein**	**MFRP**	**MFRP**		**0.03386618**	**-1.22**	**-1.25**
SL004253	Estradiol 17-beta-dehydrogenase 1	17-beta-HSD 1	HSD17B1		0.02386383	-1.21	-1.23
SL000011	alpha-Fetoprotein	AFP	AFP		0.00062497	-1.21	-1.22
**SL000521**	**Interstitial collagenase**	**MMP-1**	**MMP1**		**0.00896012**	**-1.21**	**-1.21**
SL010518	Tyrosine-protein kinase Tec	TEC	TEC		0.01272388	-1.24	-1.21
SL004938	Calcium/calmodulin-dependent protein kinase kinase 1	CaMKK alpha	CAMKK1		0.00833497	-1.26	-1.21
SL011180	Hexokinase-1	HXK1	HK1		0.003176	-1.24	-1.19
**SL009791**	**Heterogeneous nuclear ribonucleoprotein A/B**	**hnRNP A/B**	**HNRNPAB**		**0.02347986**	**-1.22**	**-1.19**
SL009341	Basigin	BASI	BSG		0.04242972	-1.19	-1.18
SL004469	Amyloid beta A4 protein	amyloid precursor protein	APP		0.01340295	-1.25	-1.16
SL007640	C-type lectin domain family 7 member A	CLC7A	CLEC7A		0.009192	-1.15	-1.16
SL005159	Erythropoietin receptor	EPO-R	EPOR		0.03180034	-1.13	-1.15
SL005224	Thrombopoietin Receptor	Thrombopoietin Receptor	MPL		0.0420125	-1.15	-1.15
SL007056	Bone morphogenetic protein 10	BMP10	BMP10		0.04172684	-1.16	-1.15
SL003974	Activated Protein C	Activated Protein C	PROC		0.02100606	-1.19	-1.15
SL007747	Serine/threonine-protein kinase TBK1	TBK1	TBK1		0.01772599	-1.15	-1.14
SL004415	Angiotensin-converting enzyme 2	ACE2	ACE2		0.02525962	-1.09	-1.14
SL020172	Relaxin receptor 1	RXFP1	RXFP1		0.01055514	-1.19	-1.14
SL007250	Phosphatidylinositol 4,5-bisphosphate 3-kinase catalytic subunit gamma isoform	PK3CG	PIK3CG		0.04439932	-1.19	-1.13
SL007888	Cystatin-S	Cystatin-S	CST4		0.04186425	-1.12	-1.13
SL001905	Mesothelin	Mesothelin	MSLN		0.01134815	-1.12	-1.13
SL018587	Immunoglobulin superfamily containing leucine-rich repeat protein 2	ISLR2	ISLR2		0.0191338	-1.24	-1.13
SL005258	Serine/threonine-protein kinase PLK1	PLK-1	PLK1		0.0282241	-1.12	-1.12
SL011588	40S ribosomal protein S3a	RS3A	RPS3A		0.02953075	-1.15	-1.12
SL009951	Protein Wnt-7a	WNT7A	WNT7A		0.03339452	-1.10	-1.12
SL000306	Brain natriuretic peptide 32	BNP-32	NPPB		0.00647505	-1.17	-1.12
SL008378	Eukaryotic translation initiation factor 4E-binding protein 2	4EBP2	EIF4EBP2		0.04397662	-1.16	-1.12
SL011708	cAMP-regulated phosphoprotein 19	ARP19	ARPP19		0.00611009	-1.18	-1.12
SL008059	40S ribosomal protein S3	RS3	RPS3		0.03730939	-1.11	-1.12
SL014209	Killer cell lectin-like receptor subfamily F member 1	KLRF1	KLRF1		0.02624641	-1.17	-1.12
SL004230	Microtubule-associated protein tau	tau	MAPT		0.03085204	-1.16	-1.11
SL014289	Killer cell immunoglobulin-like receptor 3DS1	KI3S1	KIR3DS1		0.0108866	-1.19	-1.11
SL004725	Histone acetyltransferase type B catalytic subunit	Hat1	HAT1		0.03038019	-1.17	-1.11
SL002071	Gastrin-releasing peptide	Gastrin-releasing peptide	GRP		0.02100769	-1.12	-1.11
SL005179	Interleukin-36 beta	IL-1F8	IL36B		0.01041349	-1.14	-1.11
SL000130	G2/mitotic-specific cyclin-B1	Cyclin B1	CCNB1		0.01719849	-1.18	-1.10
SL007774	Protein jagged-2	JAG2	JAG2		0.02857578	-1.12	-1.10
SL016548	AMP Kinase (alpha1beta1gamma1)	AMPK a1b1g1	PRKAA1 PRKAB1 PRKAG1		0.02868372	-1.15	-1.09
SL005308	Proteasome activator complex subunit 3	PSME3	PSME3		0.0293512	-1.15	-1.09
SL005493	Serum paraoxonase/arylesterase 1	paraoxonase 1	PON1		0.02229908	-1.13	-1.08
SL004357	Growth/differentiation factor 8	Myostatin	MSTN		0.04847629	-1.17	-1.08
SL008590	Olfactomedin-4	Olfactomedin-4	OLFM4		0.04469905	-1.14	-1.08
SL010371	Ectonucleoside triphosphate diphosphohydrolase 1	CD39	ENTPD1		0.03562801	-1.18	-1.08
SL010504	MAP kinase-activated protein kinase 5	MAPK5	MAPKAPK5		0.02438916	-1.18	-1.08
SL005392	Arylsulfatase A	Arylsulfatase A	ARSA		0.02669418	-1.11	-1.07
SL005158	C-type lectin domain family 4 member M	DC-SIGNR	CLEC4M		0.04750993	-1.18	-1.07
SL006892	Tyrosine-protein kinase ABL1	ABL1	ABL1		0.03627768	-1.11	-1.07
SL005197	Lysosome membrane protein 2	LIMP II	SCARB2		0.02261501	-1.13	-1.06
SL004625	A disintegrin and metalloproteinase with thrombospondin motifs 4	ADAMTS-4	ADAMTS4		0.00534062	-1.09	-1.06
SL009629	Methyl-CpG-binding domain protein 4	MBD4	MBD4		0.04776494	-1.10	-1.06
SL002783	Cardiotrophin-1	Cardiotrophin-1	CTF1		0.02698703	-1.07	-1.05
SL000131	Proliferating cell nuclear antigen	PCNA	PCNA		0.03904086	-1.12	-1.05
SL004299	Baculoviral IAP repeat-containing protein 7 Isoform beta	Livin B	BIRC7		0.04649281	-1.05	-1.05
SL007828	Neuregulin-4	NRG4	NRG4		0.02206438	-1.10	-1.05
SL000584	Transforming growth factor beta-1	TGF-b1	TGFB1		0.04174855	-1.07	-1.04
SL004346	Interleukin-20	IL-20	IL20		0.04854244	-1.04	-1.03
SL003990	Fibroblast growth factor receptor 2	FGFR-2	FGFR2		0.02699095	-1.11	-1.02
SL006923	TATA-box-binding protein	TBP	TBP		0.01659501	-1.05	-1.02
SL000474	Interleukin-16	IL-16	IL16		0.02977617	-1.06	-1.02
SL001713	Interleukin-17A	IL-17	IL17A		0.01722795	-1.12	-1.02
SL005165	Galectin-4	Galectin-4	LGALS4		0.04953144	-1.09	-1.02

Supplementary Table S2. Top Up-Regulated and Down-Regulated Proteins Between OSCC Cases and Healthy Controls

Increased in OSCC

**SomaId**	**Protein Name**	**Target**	**Entrez Gene Symbol**	**OSCC vs Con p-val**	**mean FC**	**median FC**
SL004296	Adenylate kinase isoenzyme 1	Myokinase, human	AK1	0.04032307	2.21	2.09
SL000836	Hemoglobin	Hemoglobin	HBA1 HBB	0.01200661	1.99	1.96
SL011073	Xaa-Pro aminopeptidase 1	XPNPEP1	XPNPEP1	0.04763114	1.90	1.79
SL008039	Alcohol dehydrogenase [NADP(+)]	AK1A1	AKR1A1	0.01399805	1.65	1.77
SL016550	Casein kinase II 2-alpha:2-beta heterotetramer	CK2-A1:B	CSNK2A1 CSNK2B	0.03407762	1.53	1.71
**SL010250**	**Stress-induced-phosphoprotein 1**	**Stress-induced-phosphoprotein 1**	**STIP1**	**0.0401434**	**1.69**	**1.61**
SL004919	Peroxiredoxin-1	Peroxiredoxin-1	PRDX1	0.0429796	1.51	1.59
SL005187	Interleukin-3 receptor subunit alpha	IL-3 Ra	IL3RA	0.03413965	1.48	1.40
**SL000521**	**Interstitial collagenase**	**MMP-1**	**MMP1**	**0.00679146**	**1.49**	**1.39**
SL007003	Desert hedgehog protein N-product	DHH	DHH	0.04786586	1.47	1.39
SL006914	Glucocorticoid receptor	Glucocorticoid receptor	NR3C1	0.02947505	1.30	1.39
SL000592	Metalloproteinase inhibitor 2	TIMP-2	TIMP2	0.02343185	1.26	1.29
**SL004457**	**Glia-derived nexin**	**Protease nexin I**	**SERPINE2**	**0.01286922**	**1.25**	**1.28**
SL003915	Kallikrein-8	kallikrein 8	KLK8	0.03721993	1.27	1.26
SL005572	Gelsolin	Gelsolin	GSN	0.04365788	1.22	1.22
SL004718	Importin subunit alpha-1	Karyopherin-a2	KPNA2	0.00433055	1.17	1.18
**SL010466**	**Membrane frizzled-related protein**	**MFRP**	**MFRP**	**0.03615788**	**1.16**	**1.17**
SL000104	Apoptosis regulator Bcl-2	Bcl-2	BCL2	0.03174288	1.12	1.16
**SL004765**	**MAP kinase-activated protein kinase 3**	**MAPKAPK3**	**MAPKAPK3**	**0.00609569**	**1.19**	**1.16**
SL008421	A disintegrin and metalloproteinase with thrombospondin motifs 1	ATS1	ADAMTS1	0.04534437	1.16	1.13
SL010393	Kremen protein 2	KREM2	KREMEN2	0.04153119	1.20	1.13

Decreased in OSCC

**SomaId**	**Protein Name**	**Target**	**Entrez Gene Symbol**	**OSCC vs Con p-val**	**mean FC**	**median FC**
SL002756	Heterogeneous nuclear ribonucleoprotein K	hnRNP K	HNRNPK	0.00780153	-1.11	-1.11
SL000319	Complement C5	C5	C5	0.04169888	-1.17	-1.11
SL003328	Complement factor I	Factor I	CFI	0.04824177	-1.18	-1.12
SL000415	Complement factor H	Factor H	CFH	0.04847355	-1.13	-1.13
SL008177	Complement component 1 Q subcomponent-binding protein, mitochondrial	C1QBP	C1QBP	0.02460562	-1.13	-1.13
SL004304	Syntaxin-1A	STX1a	STX1A	0.03672081	-1.29	-1.14
SL002565	Myc proto-oncogene protein	c-Myc	MYC	0.04640721	-1.21	-1.14
SL011069	Serine protease 27	Marapsin	PRSS27	0.03753767	-1.18	-1.15
SL001973	Mammaglobin-B	Mammaglobin 2	SCGB2A1	0.04172115	-1.16	-1.16
SL000360	Coagulation Factor X	Coagulation Factor X	F10	0.04929031	-1.21	-1.16
SL000057	Thymidine kinase, cytosolic	Thymidine kinase	TK1	0.00315418	-1.21	-1.18
**SL009207**	**Dynactin subunit 2**	**Dynactin subunit 2**	**DCTN2**	**0.0404482**	**-1.24**	**-1.20**
SL007059	Lymphocyte antigen 86	LY86	LY86	0.01685897	-1.21	-1.20
SL012148	Peroxisomal targeting signal 1 receptor	PEX5	PEX5	0.02068758	-1.17	-1.20
SL008909	Legumain	LGMN	LGMN	0.0239212	-1.19	-1.20
SL018946	Ubiquitin-conjugating enzyme E2 G2	UB2G2	UBE2G2	0.01847193	-1.19	-1.21
SL003220	C3a anaphylatoxin des Arginine	C3adesArg	C3	0.00040137	-1.20	-1.22
**SL000309**	**Complement C1q subcomponent**	**C1q**	**C1QA C1QB C1QC**	**0.00816457**	**-1.28**	**-1.23**
SL004183	Cadherin-3	P-Cadherin	CDH3	0.04001921	-1.25	-1.24
SL002561	Parathyroid hormone-related protein	PTHrP	PTHLH	0.0010905	-1.23	-1.24
SL004865	Cadherin-6	Cadherin-6	CDH6	0.01746239	-1.30	-1.24
SL020171	HERV-H LTR-associating protein 2	HHLA2	HHLA2	0.0252974	-1.33	-1.25
SL010379	Semaphorin-3A	Semaphorin 3A	SEMA3A	0.0023193	-1.29	-1.26
SL006993	Mitogen-activated protein kinase 13	MK13	MAPK13	0.02080795	-1.21	-1.26
SL000414	Complement factor B	Factor B	CFB	0.02846216	-1.19	-1.26
**SL000268**	**Angiostatin**	**Angiostatin**	**PLG**	**0.00396009**	**-1.30**	**-1.27**
SL005234	Growth/differentiation factor 9	GDF-9	GDF9	0.02368223	-1.21	-1.27
**SL000586**	**Thrombin**	**Thrombin**	**F2**	**0.0339196**	**-1.33**	**-1.28**
SL000280	Aspartate aminotransferase, cytoplasmic	GOT1	GOT1	0.01697681	-1.32	-1.30
SL004591	Granulocyte colony-stimulating factor receptor	G-CSF-R	CSF3R	0.00233852	-1.32	-1.30
SL004060	Endothelin-converting enzyme 1	Endothelin-converting enzyme 1	ECE1	0.00119138	-1.34	-1.31
**SL007173**	**Granulins**	**GRN**	**GRN**	**0.03783666**	**-1.25**	**-1.33**
**SL010328**	**Mediator of RNA polymerase II transcription subunit 1**	**MED-1**	**MED1**	**0.0172352**	**-1.29**	**-1.34**
SL009213	Lysosomal protective protein	Cathepsin A	CTSA	0.02722162	-1.34	-1.37
SL014228	SLAM family member 6	SLAF6	SLAMF6	0.02599882	-1.42	-1.42
**SL007336**	**Protein SET**	**SET**	**SET**	**0.01997398**	**-1.40**	**-1.44**
SL004588	Interleukin-1 Receptor accessory protein	IL-1 R AcP	IL1RAP	0.01508365	-1.54	-1.57
SL006512	Hepatocyte growth factor activator	HGFA	HGFAC	0.03967619	-1.63	-1.60
SL011769	Acidic leucine-rich nuclear phosphoprotein 32 family member B	AN32B	ANP32B	0.02529884	-1.51	-1.66
SL005675	ATP synthase subunit beta, mitochondrial	ATP synthase beta chain	ATP5B	0.03529201	-1.85	-2.28
SL019096	E3 SUMO-protein ligase PIAS4	PIAS4	PIAS4	0.04046524	-2.35	-2.93
SL000572	Serum amyloid A-1 protein	SAA	SAA1	0.00352611	-3.87	-3.89
